# Quality of life from women’s perspective in the exercise of sex work: a study of social representations

**DOI:** 10.1590/0034-7167-2022-0169

**Published:** 2023-05-29

**Authors:** Pablo Luiz Santos Couto, Maria Luisa Pereira Neves, Luiz Carlos Moraes França, Antônio Marcos Tosoli Gomes, Samantha Souza da Costa Pereira, Alba Benemérita Alves Vilela, Dejeane de Oliveira Silva, Sérgio Correia Marques

**Affiliations:** I Universidade Estadual do Sudoeste da Bahia. Jequié, Bahia, Brazil; II Centro Universitário FG. Guanambi, Bahia, Brazil; III Universidade do Estado do Rio de Janeiro. Rio de Janeiro, Rio de Janeiro, Brazil; IV Universidade Estadual de Montes Claros. Montes Claros, Minas Gerais, Brazil; V Universidade Estadual de Santa Cruz. Ilhéus, Bahia, Brazil

**Keywords:** Quality of Life, Sex Workers, Sex Work, Women’s Health, Nursing, Calidad de Vida, Trabajadores Sexuales, Trabajo Sexual, Salud de la Mujer, Enfermería, Qualidade de Vida, Profissionais do Sexo, Trabalho Sexual, Saúde da Mulher, Enfermagem

## Abstract

**Objectives:**

to analyze the social representations elaborated by sex workers from Alto Sertão Produtivo Baiano about quality of life.

**Methods:**

a qualitative study, based on the Social Representation Theory, carried out in the region of Alto Sertão Produtivo Baiano, with 30 sex workers. Individual in-depth interview was carried out, with speeches organized in a corpus and treated in IRAMUTEQ, enabling lexical analysis for Descending Hierarchical Classification.

**Results:**

four thematic classes emerged, in which social representations of quality of life pervade: money earned to supply needs; association with healthy living and obtaining health (physical and mental); balance of emotions (although there are some negative sensations such as fear and anxiety); and faith in a deity.

**Final Considerations:**

the social representations elaborated by sex workers about quality of life are anchored in concepts, subjective and practical, punctuated by the World Health Organization.

## RESUMO

**Objetivos:**

analisar as representações sociais elaboradas por trabalhadoras sexuais procedentes do Alto Sertão Produtivo Baiano sobre qualidade de vida.

**Métodos:**

estudo qualitativo, baseado na Teoria das Representações Sociais, realizado na Região do Alto Sertão Produtivo Baiano, com 30 trabalhadoras sexuais. Realizou-se entrevista em profundidade individual, com discursos organizados em um *corpus* e tratados no *software* IRAMUTEQ, possibilitando a análise lexical para a Classificação Hierárquica Descendente.

**Resultados:**

revelaram-se quatro classes temáticas, nas quais as representações sociais da qualidade de vida perpassam: pelo dinheiro conquistado para suprimento das necessidades; pela associação à vida saudável e obtenção da saúde (física e mental); pelo equilíbrio das emoções (ainda que haja algumas sensações negativas como o medo e ansiedade); e pela fé em uma divindade.

**Considerações Finais:**

as representações sociais elaboradas pelas trabalhadoras sexuais acerca da qualidade de vida estão ancoradas em conceitos, subjetivos e práticos, pontuados pela Organização Mundial da Saúde.

## RESUMEN

**Objetivos:**

analizar las representaciones sociales elaboradas por trabajadoras sexuales del Alto Sertão Produtivo Baiano sobre la calidad de vida.

**Métodos:**

estudio cualitativo, basado en la Teoría de las Representaciones Sociales, realizado en la región de Alto Sertão Produtivo Baiano, con 30 trabajadoras sexuales. Entrevista individual en profundidad, con discursos organizados en un corpus y tratados en IRAMUTEQ, posibilitando el análisis léxico para Clasificación Jerárquica Descendente.

**Resultados:**

surgieron cuatro clases temáticas, en las que impregnan las representaciones sociales de la calidad de vida: el dinero ganado para suplir las necesidades; para la asociación con la vida sana y la obtención de la salud (física y mental); el equilibrio de las emociones (aunque hay algunas sensaciones negativas como el miedo y la ansiedad); y la fe en una deidad.

**Consideraciones Finales:**

las representaciones sociales elaboradas por las trabajadoras sexuales sobre la calidad de vida están ancladas en conceptos, subjetivos y prácticos, puntuados por la Organización Mundial de la Salud.

## INTRODUCTION

Sex work has been understood as a trade in the body itself, whose service is common and offered for the sexual satisfaction of countless people, especially men. According to the Ministry of Labor and Employment, many women who offer this type of service experience the freedom of their bodies to obtain profit and income, when serving and accompanying customers, as they break with the sociocultural determinism of female sexuality, as they do not restrict sexual practice to the private space of marriage^(1-2)^.

In this sense, the practice of prostitution by women has been common throughout history, since the period before the formation of cities and social structures, based on patriarchy and conservative religious doctrines (when marriage was established with strict marriage rules, to establish control over women’s bodies and sexuality), in order to obtain financial income for support^(3-7)^. Sex work is considered the concept proposed by progressive feminist theorists, which defines it as a work activity in which the practice of sex is consensual and remunerated, in which women offer sexual pleasure (to customers) in exchange for income that enables them to provide for their families^(8)^.

Thus, when looking at the sexual service performed by women, one must break with common sense and fundamentalist and stigmatizing positions as well as with the prejudice and judgment of value perpetrated against the profession^(2-8)^. Such factors foster the various social inequities suffered by sex workers (sex, race and class), which are built by the patriarchal system of countries that do not have this legal work activity, cooperating for the invisibility of the profession by the State and maintenance of situations of vulnerability^(3-5)^.

Constructed stigmas tend to compromise the well-being, health, relationships and, consequently, the quality of life of sex workers, since a considerable portion makes up the base of society’s pyramid, with difficulties in accessing minimum and basic services for survival^(9-10)^. Therefore, to consider that sexual service is a profession that only provides pleasure is to contribute to maintenance of social exclusion of people who do not have their profession regulated, compromising assessment and representation of quality of life^(1,6,9)^.

The notion of quality of life (QoL) is broad and marked by subjectivity, whose concept goes beyond biologist, positivist and reductionist considerations, not just the presence of disease or absence of health. According to the World Health Organization (WHO), it is marked by affective, psychological, emotional, attitudinal, political, cultural and social issues, as well as everything that hinders the maintenance of human rights^(11)^.

In this perspective, the Social Representation Theory (SRT) is used to support studies whose aim is to deepen meanings and knowledge that groups of social belonging build in relation to subjective phenomena, since knowledge is elaborated and shared among people, with a practical object. Social representations (SR) are socially constructed by people who share (or do not) consensus, such as sex workers^(2,12)^.

Some previous studies have been published involving QoL with the contribution of SRT, with people living with HIV^(11)^, trans women^(13)^ and older adults^(14)^, for instance. However, there was no publication that brought the SR of QoL for sex workers.

Thus, considering that SR are built in a dynamic process of sharing information in a sociocultural group about an object or phenomenon, based on experiences in a given context, it is relevant to go deeper into how these women represent QoL. Moreover, as the context is also decisive for the construction of SR, it is considered that the region of *Alto Sertão Productivo Baiano* has in its socio-historical constitution marked intersectional iniquities, as influence of patriarchy, social inequality and lack of basic goods to obtain QoL, with the increase of poverty. Added to this justification is the scarcity of scientific research on the object presented in interface to SRT. In view of this, the following guiding question was drawn: what are the SR that sex workers from *Alto Sertão Produtivo Baiano* have about QoL in the daily life of sexual service?

## OBJECTIVES

To analyze the SR elaborated by sex workers from *Alto Sertão Produtivo Baiano* on QoL.

## METHODS

### Ethical aspects

The principles and national and international standards of ethics in research involving human beings of the Brazilian National Health Council Resolution 466/2012 were respected, with the project submitted and approved by the Research Ethics Committee. All participants signed the Informed Consent Form. In order to guarantee secrecy and preserve the anonymity of each one, codes were adopted to name the participants, using the letter “I” for interviewee, followed by a number (e.g., I1).

### Theoretical-methodological framework

SRT is a common sense theory, designed to give meaning and elucidation to ideas, concepts and meanings in instances of socially constructed practical knowledge, considering the shared symbolic aspects as the origin of representational processes^(12)^. Thus, representation takes on meaning from that knowledge formed through a collective sense, in exchanges and sharing of information and intragroup experiences, with repercussions in practices and behaviors constructed and socially disseminated^(15-16)^.

The theory’s procedural perspective, adopted for this study, refers to the formation of social representations from the social memory and construction of meanings, anchored and objectified in the form of ideas, ideologies, symbols, behaviors and attitudes. Anchoring and objectification are two mechanisms of the mental system, through memory and past conclusions, transforming what is unknown, disturbing and strange, into known and familiar^(12)^.

### Study design

This is a qualitative study, based on SRT’s procedural perspective, developed with 30 sex workers from *Alto Sertão Produtivo Baiano*. During the study development and instrumentalization, the authors complied with the norms and criteria of quality rigor, as they were guided by the Consolidated criteria for REporting Qualitative research (COREQ).

### Methodological procedures, study setting and data sources

The women who contributed to the study are from *Alto Sertão Produtivo Baiano*, covering 19 municipalities, with about 400,000 inhabitants^(2)^, a region in which they attend or live, reside and/or perform sexual service.

As selection and recruitment criteria, being over 18 years old and having been involved in the sexual service for at least 1 year (considering that experience allows for a broader view of the sexual service) were adopted. Thus, 30 female workers contributed to the study, answering the instruments, among the 39 invited. There was no need to apply exclusion criteria, because participants received invitations and only those who accepted contributed as the study.

The convenience sample was delimited using the snowball technique, with the help of Community Health Workers (CHW) who work in the neighborhoods and regions where the workers are present, who indicated and contacted the first participant, which was indicating some others that, in turn, indicated the others. Snowball is a technique of selection and recruitment of participants, whose population’s access and estimation are difficult, such as sex workers^(17)^.

### Data collection and organization

Data collection was carried out by three researchers responsible for the study (two master professors and a monitor from the research group responsible for data collection, who was previously trained). It took place individually between April 2017 and June 2018, in rooms reserved in Family Health Strategies of neighborhoods where several “businesses” are located, such as bars, restaurants, pensions and inns, in which sex workers work. Participants, who did not reside in the host city, scheduled visits to the spaces for the services developed by them, with the help of CHW, who work in the assigned territory. It should be noted that approaching participants took place before collection, still with the extension project of health promotion with sex workers in the region and also in partnership with the local Testing and Counseling Center.

A script with structured items was used to characterize participants, as well as two questions that guided the in-depth interview, a technique adopted for data collection: tell me what you understand by QoL and how you perceive QoL in your day to day in the sexual service? Furthermore, with the unfolding of the interviews, and in order to obtain greater clarity in the answers, they were asked about “what strategies they used to achieve well-being and QoL in a work service permeated by vulnerabilities”. The interviews lasted about 25 to 30 minutes. The responses were recorded with audio resources on a cell phone, then transcribed in full in Microsoft Word 2016, on the same day that the interviews ended.

### Data analysis

The transcribed speeches were carefully read; then, they were transformed into a text corpus, being developed the lexical analysis with the help of IRAMUTEQ (*Interface of R pour les Analyses Multidimensionnelles de Textes et de Questionnaires*)^(15)^. IRAMUTEQ issues a class dendrogram that allows performing statistical analyzes on qualitative variables, through lexicography of the Descending Hierarchical Classification method (DHC), obtaining contexts and classes generated on text segments (TS) arising from the lexicons and their sets of words, presented in reduced forms, from matrices of text corpus, crossing TS and words in continuous chi-square tests (X^2^)^(17-18)^. This DHC resource presents responses divided into TS, which originate the thematic classes^(18)^.

## RESULTS

Among the 30 participants who composed the sample of this study, the majority were aged between 18 and 35 years (78.26%); had few years of schooling, five years or less of study (53.62%); self-declared black (59.42%); self-reported being Catholic (45.07%) and Protestant (34.6%); worked for less than 05 years (68.12%); was not satisfied with the profession (55.97%); used condoms during sexual intercourse (63.77%); and reported using hormonal oral contraceptives (66.66%). It should be noted that, due to unfavorable sociodemographic conditions, and in line with low educational level, it was evident that the majority have a variable fixed monthly income, in which 67% charge between R$30.00 and R$100.00 (about US$6.00 and US$20.00, respectively) per program, monthly, reaching a maximum of just over one minimum wage. The majority, 91%, declared themselves to be providers, i.e., their children, spouses and other relatives survive on income from sexual services.

**Figure 1:**
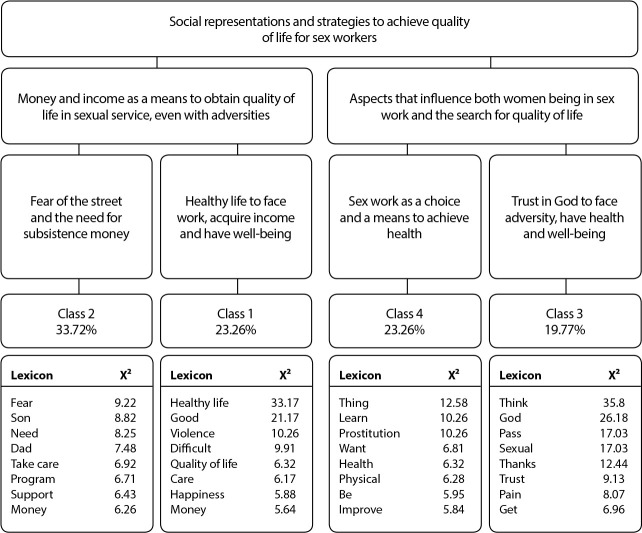
Class dendrogram for Descending Hierarchical Classification, Guanambi, Bahia, Brazil (N=30)

The DHC dendrogram (Figure 1) generated four classes, from 2,126 TS extracted from the interviews, with 85.32% of use of analyzed text corpus, which enables a better understanding of words and phrases spoken by participants, presented in elementary context units (ECU).

The classes arising from analysis bring the elements that have a statistically significant influence that make SR attributed to QoL meaningful, showing the greatest strengths in relation to the frequency with the central and most important word for the representation of sex workers. The classes were named according to lexicons of greater X^2^, and emerged and were organized with the dendrogram processing in relation to oppositions with other generated classes, allowing access to the construction of an explanatory system about the phenomenon/object investigated.

For a better understanding of this structure of textual division, which forms the corpus of answers, to reach the classes, there is presentation of semantic-lexical contents that are heterogeneous, because they have successive divisions until obtaining class homogeneity, as shown in the dendrogram (Figure 1). There are two large thematic chunks and four classes, namely: the first referring to classes 2 and 1, the second, to classes 3 and 4. The classes presented in Figure 1, according to their theme and the content that composes each one of them, are described in the following.

### Class 2 - Fear of the street and the need for subsistence money

Class 2 has 33.72% of ECU, having fear (x^2^ = 9.22), child (x^2^ = 8.82), need (x^2^ = 8.25), program (x^2^ = 6.11), support (x^2^ = 6.11), among other elements, as the main elements that compose it.

*Since I separated from my husband, I have seen no other option but to prostitute myself and, since then, I decided that I would only have sex for money and not for pleasure*. (I17)

*Quality of life being well with yourself, being healthy, having dignity, being able to pay a doctor, if you are feeling pain, buy the medicine you need, take care of your body, go to the gym*. (I9)

*But not always possible, and then comes the sadness, having to face the fear of the dangers on the street, for someone to do something, for us to feel like garbage*. (I23)

*Coming home even if your son doesn’t know about your life, but you know that his love is yours alone. It makes up for the awful, stressful things that happen to us*. (I16)

A process of SR formation about the activity performed is perceived as a way to achieve QoL, even if the secrecy about the profession is maintained in front of family members. Fear may be associated with the apprehension of being victims of violence and/or the prejudice suffered in society and the lack of formulation and implementation of health policies aimed at protection, care and safety.

### Class 1 - Healthy life to face work, acquire income and have well-being

This class is responsible for 23.26% of ECU, composed of terms healthy life (x^2^=33.17), good (x^2^=21.17), violence (x^2^=10.26), difficult (x^2^=9, 91) and QoL (x^2^=6.32). There are different facets present in the profession’s routine, in addition to elements that narrate the reflexes in their lives.

*Self-love, comfort, helping the family. Money, peace, security, love, prevention, opportunity, financial life, healthy life, work, comfort. I think my life is good, I just need a little more health*. (I15)

*It’s having a financial life, good opportunities in our day, finding a good customer. To lead a healthy life is to be healthy to work and be comfortable. The profession is not pleasant. Emotionally difficult*. (I10)

*Quality of life is having the love of your family, having the assurance that you will not suffer any harm or violence. Money that gives you a secure financial life and guarantees enough for you to have mental health without stress and anxiety and lead a healthy life*. (I20)

*Having happiness, leading a healthy life, which does not always happen. Having security even knowing that this is difficult, because we are afraid and are subject to violence from the street to rape, quality of life comes from within, it also comes from the vanity of self-love*. (I 1)

The SR verified in the ECU permeate aspects of emotions and feelings that lead to mental health, such as anxiety, happiness, fear, vanity and self-love. In turn, the practical perspective refers to adoption of a healthy life, comfort, the need to have security to face violence. Finally, it is clear that money is still linked to this class, due to the importance of obtaining income for self-promotion of QoL in SR.

### Class 4 - Sex work as a choice and a means to achieve health

This class shows 23.26% of ECU used, whose main lexicons with greater x^2^ are thing (x^2^=12.58), learning (x^2^ = 10.26), prostitution (x^2^=10.26), want (x^2^=6.81) and health (x^2^ = 6.32). Such terms may be associated with the need for them to readjust everyday life, due to the stigmas ingrained in this work practice.

*That fear I had of being violated, of being humiliated by customers, it was at the beginning when I was on point. Once I went to file a police report and the police chief said it wasn’t enough, because of the life I started. So, I became disillusioned and tried to stay in places that gave me more protection, like the house of a lady who rents out a room for us.* (I22)

*Choosing customers better, everything has improved. I learned to do things right, but sometimes I still feel empty when I’m at my house taking care of it, doing the laundry, but then I forget about the bad things*. (I7)

*If someone asks something, I don’t really tell them what I do, because I’m very young and work with prostitution, I could study, so the fact of living in hiding harms my emotions*. (I26)

*How do I tell you? It was an initiative, my choice to be in prostitution. I entered this life to take care of my health and, for me, today, it is one hundred percent, although in the condition of this service we encounter many problems, such as violence*. (I22)

*Quality of life and health go together. You know, if I don’t have health, lead a healthy life, I won’t be well at work, in addition to the absence of diseases, it’s a person’s physical mental social state*. (I2)

It is considered that, in this class, paid sexual service, practiced by women, is a professional choice and, even with the problems faced in daily work, it is through income that they manage to find ways to access material goods and health services, even if the secret about work activity is kept. Here, QoL is represented in the idea of health (physical and mental) and self-care to achieve self-esteem.

### Class 3 - Trust in God to face adversity, have health and well-being

This class obtained 19.77% of ECU, bringing elements think (x^2^ = 35.8), God (x^2^ = 26.18), go through (x^2^ = 17.03), sexual (x^2^ = 17.03) and pain (x^2^ = 8.07). This class talks about the relationship with professionals’ self-esteem and health condition, the search for improvement based on this condition and the connection with the transcendent, here identified as God.

*It was thinking about this that my intimate life was going to improve my comfort, my home and, thank God, everything got better when I started to love myself more and have self-esteem*. (I11)

[...] *I trust God a lot, no matter how great the tribulation, I put God in front. The money I work as a call girl, I wouldn’t earn anywhere, in any madam’s house.* (I23)

*It also comes from within, from the faith we must have* [...] *a healthy life, when you have money, it’s easier* [...] *I pay for procedures for our body, in addition to working out when I have time.* (I24)

*I was already raped by an uncle of mine, when I was a girl, so when a man touches me, I get anxious, but I swallow hard and go, because I need the money, to take what my family needs.* (I3)

This last class presents the representation of QoL referred to the belief in a deity (God) as a way to conquer happiness and well-being, in the midst of a stigmatized work practice.

## DISCUSSION

The characteristics of sex workers’ profile studied here were similar to research carried out in the past either in Brazil, Malaysia, or Africa (Kenya), with a predominance of women with a low educational level, self-declared black and part of them not satisfied with the profession. Moreover, they obtain a relatively low income, to be able to meet the demands of family members who live with them^(2,4,10,19)^.

For dissatisfaction with sex work, represented by some women who are confident of receiving some remuneration in this profession, results of previous studies carried out in Belo Horizonte (Brazil), France and Canada, revealed that there is difficulty for sex workers to access social rights, such as labor and social security, due to State negligence, reverberated in invisibility and lack of protection. These studies also reinforced that health professionals perpetuate the stigma of this work, through institutional prejudice^(4,8,20)^.

In these contexts that reveal the profile, struggles and confrontations for better conditions for the practice of sexual service, representational meanings for QoL are constructed in these women’s cognition systems, which occurs in a collective movement of exchange of information, knowledge and intragroup experiences, enabling interpretation of reality, common sense and practices developed^(2,11-12,15)^.

These first SR attributed to QoL reveal the income from the sexual service to face adversities, being the SR practical content, as they show themselves as a phenomenon that justifies the construction of consensual ideas, identified and shared by the social actors, providing a global view of their context^(12)^.

The particularity of being a woman, sex worker and, often, black and poor, causes insecurity to carry out their activities, considering the fear of being violated and judged guilty, crushing the State’s role of victim, linked to oblivion and impunity for their aggressors^(4-6,21-22)^. This condition is reflected in the high rate of femicide in Brazil, where the fact of being a woman is enough to justify the behavior of “men” and their “good customs”, structured from common sense, the pejorative ideal of the female figure, especially if it is linked to some scenario of expression of sexuality^(1,3-4)^.

This SR about the basic rights of any citizen, which are enshrined in the constitution, processed in intragroup exchange in society, shows the autonomy that many sex workers have when reinforcing their opinions, experiences, emotions and all the value systems that contribute to the notoriety of the representation about a phenomenon and, here, QoL. The association with the positive or negative sensations present in SR serves to understand vulnerabilities and confrontations of the health-disease process in relation to the subjectivity that the social group presents^(23-24)^.

It is considered that SR formation is supported by common sense knowledge and not by technical-scientific and theoretical knowledge, especially when the social group is formed by people with such marked vulnerabilities, such as sex workers, since it originates from everyday experiences, serving as a guide and reading of reality, functioning as a language due to its symbolic function^(12,15)^.

Thus, if, on the one hand, there are people who relate to greater access to health services, on the other hand, there are some who condition psychosocial-emotional well-being. Moreover, many others consider the acquisition of money as a preponderant factor for the conquest of goods and access to different sectors of society^(2)^. Results of previous studies, carried out with sex workers from France, Africa and Brazil, revealed the importance that money has, above all due to the possibility of acquiring goods^(2,8,19)^. From this perspective, the SR elaborated by this segmented group of women, in the face of socially shaped convictions, will designate both the QoL SR and the notion of such a term in their daily work^(15,24)^.

Even so, the presence of negative feelings, such as fear and apprehension in the SR, refers to the context presented in sex work’s daily life, such as the violence sometimes perpetrated by customers or exploiters, the difficulty in discussing and guaranteeing using condoms with the men who pay for the service, the uncertainty of having a good income every day of work, in addition to constant prejudices and stigmas, very common in various services and sectors^(2,4-5)^.

There is a negative connotation to QoL in participants’ SR. Even constituting a minority, they verbalized affective-emotional lexicons, such as sadness, stress and anxiety, revealing that happiness is a feeling somewhat distant from the reality experienced by them. Such representations can be verified in the results of other studies, which associated the conditions of the profession and life of many of them with physical and psychological abuse and submission to men^(25-26)^.

Thus, several professionals reported that the act of performing the sexual service is linked to the difficulties they face on a daily basis, namely: economic, family, integration into the formal job market, few years of study and affective aspects. This issue is reaffirmed by a study carried out in Iran, which reported the deficit of opportunities distributed by state power, resulting in a state of extreme poverty, being a preponderant factor for the search for subsistence with sex work^(8,25,27)^.

These situations corroborate the scientific evidence described in a study, carried out in Mexico, on the accountability of women in the context of violence intended for them, with the media world contributing to the construction of this paradigm, by reporting such information. This condition reflects in prejudiced and misogynistic SR of sex work, dictating that such workers flee the ideal normative standard, just by linking sex to money, making the profession associated with contempt and abomination^(28)^.

Sexual autonomy and over one’s body (in relation to sexual practice outside of marriage) shocks society, governed by the culture of patriarchy, since paid sexual practice, both in Brazil and in France, establishes an economic exchange with pleasure/sexual^(2,8)^. Determining the time and sexual service to be offered, as well as agreement regarding the stipulated value, leads to disruption of historical-cultural constructs demarcated for women, because, even if many are exploited, many others are directly responsible for the work performed^(1-3,5)^. It is with this income that many obtain financial independence, comfort, acquisition of material goods, care practices to achieve a pleasant appearance for customers and promotion of a healthy and dignified life for themselves and their families^(2,8,25)^.

Other terms revealed in the QoL SR concatenate the idea of physical and mental health, self-esteem, love and care, once again supporting the notion of the QoL concept. Adopting healthy life habits and, therefore, trying to lead a healthy life, may raise QoL, even if it is difficult at times, due to the change in sleep and rest pattern and not always being able to do physical activity, in addition to the involvement of some with alcohol, tobacco and/or other illicit drugs^(9,26-27)^.

One point should be highlighted, as it was present in SR identified with classes 2 and 4 the need for part of them to maintain the secrecy of their work activity, in the face of their social circle, such as family and friends. Possibly, such evidence is related to absorption of social stigmas attributed to them, since some are in this work activity to obtain it of their own free will; otherwise, many others do not see any other form of work activity and, therefore, do not experience the freedom and autonomy evidenced in previous studies^(1-3,5,8)^. This decision can foster invisibility and make it difficult to implement/guarantee rights as citizens and working class, as well as the social recognition of this activity as a profession, in the sense of being inserted in the class’s social movements that are in continuous struggle for rights^(3,6,9,23,29)^.

In one of Ivana Marková’s postulates about SR, there is the idea that the “self” and the “others” (Ego-Alter), through interdependence and interaction, have the possibility of jointly creating a social reality permeated by objects of beliefs, knowledge, images, or symbols that give meanings and meanings to a phenomenon, determined by their social experiences, their intentions, expectations and understanding of a situation^(15)^. In this regard, possibly, the sex workers studied here conform their SR on QoL through articulation as a class organization, for survival in the face of the problems present in sexual service as well as a subterfuge for state invisibility.

By revealing, in the representations, faith in a superior being (God) as a subjective instrument to achieve balance, well-being and health, the importance that belief in a deity has for the QoL of this group of women is demonstrated^(11)^. People who daily experience marginality and experience intersectional vulnerabilities, enhanced by the absence of State support, resort to this subterfuge to face daily obstacles, which make the practice of sexual service difficult^(4,7,25-26,30)^. Previous studies carried out in Brazil, such as in Belo Horizonte, in the *Alto Sertão Produtivo Baiano*, as well as in the Amazonian border with Colombia and Peru, show the importance of belief in God as a means to balance emotions and maintain mental health^(2,26,29)^.

The SR of QoL associated with a healthy life also involves obtaining financial resources, which is essential for them to maintain care for their bodies in their entirety, considering them to be holistic beings. The money acquired from this occupation also pays for the maintenance of their family nucleus^(5,8)^. In addition to this, in line with the results of previous studies, it is possibly with the income from the exercise of sex work that they access private health services, to keep meeting their health demands without necessarily exposing their work activity^(1-2)^.

Furthermore, it should be noted that, in the SR of the group of women in question, it was present that health care can refer to the concern with the body physical appearance and maintenance that meets required standards, for women in society, governed and guided by the macho and capitalist culture: slim silhouette, with a healthy appearance, preferably with defined body contours (toned buttocks and leg muscles). It is also another need imposed on women who work in sex work so that they have more possibilities to offer their services to men who tend to pay ‘more’^(1,3-5,8)^. This may be another facet of capitalism, when they have in SR of QoL a relationship between income and body health maintenance, not necessarily with the specificity of prostitution itself, but rather as a characteristic aimed at the context of work in the capitalist world^(5)^.

This study brought to light SR of sex workers from the upper productive hinterland of Bahia on QoL, associated with money/income as a way of obtaining a healthy life, security, mental health, protection, care and self-love. Health is a determining factor for work performance, as they bring self-care, emotional and spiritual balance in SR, limits imposed for maintaining intimacy, since, in the capitalist work world, there is a demand for healthy professionals to maintain production^(2,5,8)^.

### Study limitations

The limiting factors were restrictions of the study universe that allowed presenting the SR of a segmented population group, associated with a single context, among the many existing in Brazil. The object of study involves a theme permeated with stigmas and prejudices, which is sexual service, performed by women, making it impossible for more women to participate, due to shame or fear of exposure. Therefore, it is suggested that new research be carried out that transversalize SRT, QoL and sexual service in other regions of the country, in order to broaden discussions about the study.

### Contributions to nursing, health, or public policies

The results will enable health professionals, especially nurses, to rethink and reflect on the importance of providing humanized, welcoming, empathetic care, anchored in active therapeutic listening, free of judgments, prejudices, stigmas and discrimination, based on the interest in constantly updating the demands arising from women inserted in the sexual service. Reflections on the care provided to sex workers should focus on actions based on promoting dignity so that the care provided is universal, comprehensive and equitable.

## FINAL CONSIDERATIONS

Sex workers revealed in their SR that QoL permeates the money earned from the sexual service, as it provides the means to acquire material goods and support themselves and their families, aspects that make up the understanding they have about essential items to obtain well-being and a healthy life. According to the representational meanings present in the speeches, having a healthy life, balancing emotions and positive feelings, as well as safety in the profession’s daily living, refer to the theoretical outline of QoL presented by the WHO. The search for the divine and the belief in God were linked to the notion of QoL and its reach, as it is a means of having emotions and feelings harmonized with the inner “self”, fundamental for facing situations of vulnerability.
